# Fabrication and Functionalisation of Nanocarbon‐Based Field‐Effect Transistor Biosensors

**DOI:** 10.1002/cbic.202200282

**Published:** 2022-11-03

**Authors:** Chang‐Seuk Lee, Rebecca E. A. Gwyther, Mark Freeley, Dafydd Jones, Matteo Palma

**Affiliations:** ^1^ Department of Chemistry School of Physical and Chemical Sciences Queen Mary University of London Mile End Road London E1 4NS UK; ^2^ Molecular Biosciences Division, School of Biosciences Cardiff University Cardiff CF10 3AX UK

**Keywords:** biosensors, carbon nanotubes, field-effect transistors, graphene, point-of-care diagnostics, surface functionalisation

## Abstract

Nanocarbon‐based field‐effect transistor (NC‐FET) biosensors are at the forefront of future diagnostic technology. By integrating biological molecules with electrically conducting carbon‐based platforms, high sensitivity real‐time multiplexed sensing is possible. Combined with their small footprint, portability, ease of use, and label‐free sensing mechanisms, NC‐FETs are prime candidates for the rapidly expanding areas of point‐of‐care testing, environmental monitoring and biosensing as a whole. In this review we provide an overview of the basic operational mechanisms behind NC‐FETs, synthesis and fabrication of FET devices, and developments in functionalisation strategies for biosensing applications.

## Graphical abstract

**Next‐generation diagnostics**: Nanocarbon‐based field effect transistors (NC‐FETs) use incoming target analytes to impart a gating effect on conductance. Here, we review the operational mechanisms behind NC‐FETs, the fabrication and functionalisation of NC‐FETs with receptor biomolecules and downstream biosensing applications.

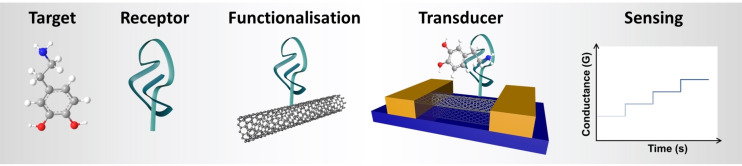

## 1 Introduction

### 1.1 What are nanocarbon‐based field‐effect transistor biosensors

Advances in biosensing applications into the nanoscale realm is now becoming a reality. Increased understanding of nanomaterial electrical properties and the use of new nanotechnology‐techniques allows researchers to now overcome some of the previous limitations of macroscale electronic devices, such as thermal degradation, short lifetime, and size/bulkiness.

**Figure 1:**
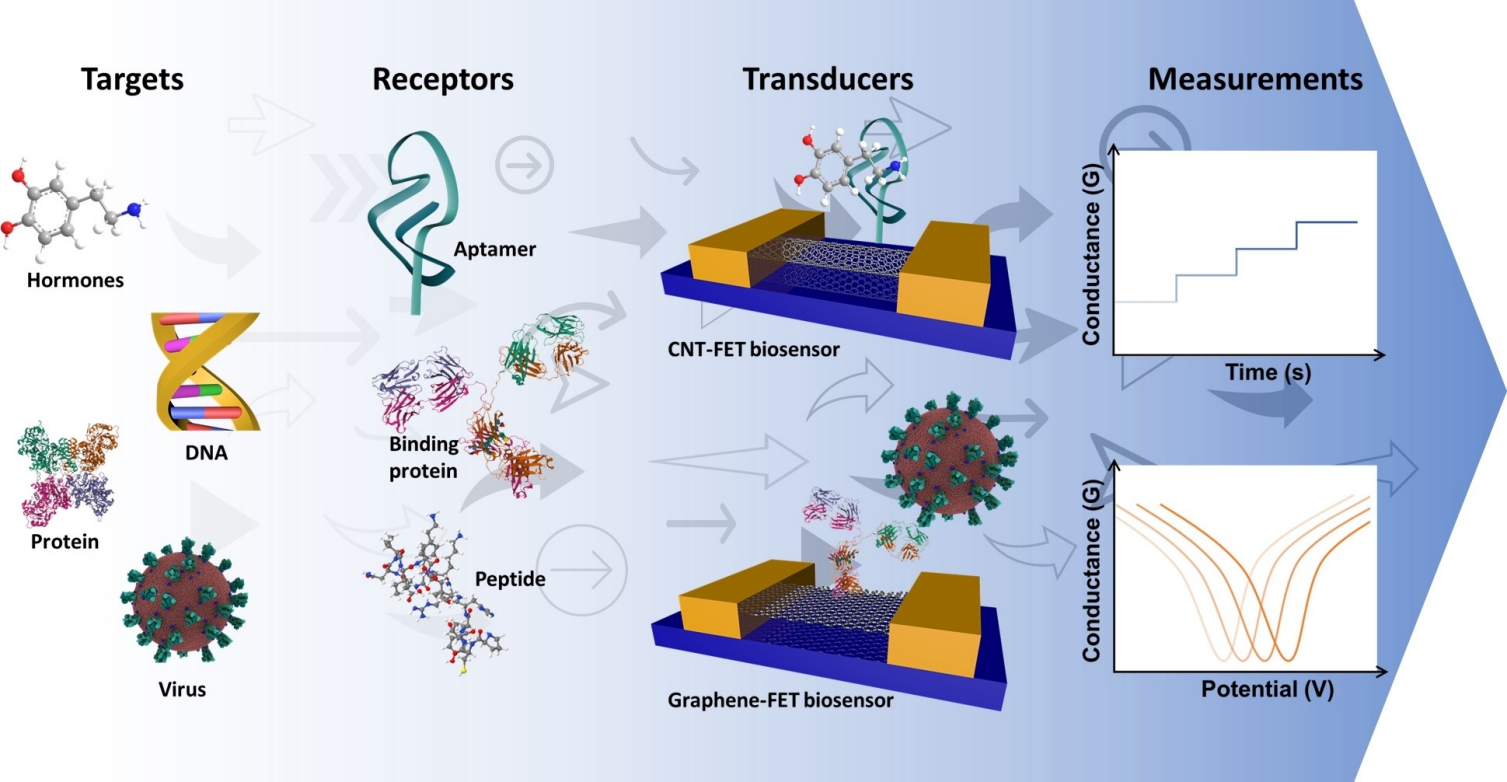
Overview of FET biosensor setups. Targets can range from large macromolecular systems such as viruses to complex biomolecules such as proteins and DNA to small molecules (e. g., hormones). The receptors for the targets include nucleic acid aptamers, binding proteins (e. g., antibodies) and peptides. Nanocarbon materials are placed between electrodes and the receptor attached. Signal transduction is then achieved though binding of the target (via the receptor) to generate a change in electrical signal.

The two key components of an electrical biosensor are the receptor and transducer. The receptor identifies the target biomolecule through formation of complementary, mainly non‐covalent interactions.[^1^, ^2^, ^3^, ^4^] Receptors routinely take the form of proteins (e. g., binding proteins such as antibodies) or nucleic acids (e. g., DNA aptamers). The role of the transducer is to convert the biological binding event into a measurable electrical signal (changes in current, conductance, resistance, etc.).[^5^, ^6^, ^7^] Nanocarbon‐based field‐effect transistor (NC‐FET) biosensors are electronic platforms that use carbon nanomaterials such as carbon nanotubes (CNTs) and graphene as the transduction material (Figure 1). Carbon‐based nanomaterials have received increasing attention as the basis for nanoscale biosensing platforms due to the size, biocompatibility, high charge carrier mobility, and sensitivity to changes in local environment.[^8^, ^9^, ^10^, ^11^] CNTs and graphene are the two most widely used materials to fabricate NC‐FET biosensors owing to their intrinsic properties including ballistic electron transport, large surface area to volume ratio, facile functionalisation strategies, accessibility to synthesis, and more.[^12^, ^13^, ^14^]

### 1.2 Advantages of using NC‐FET compared to previous electrical devices

Electrical biosensors often employ a field‐effect transistor (FETs) setup as a transducer. A FET setup has several advantages including label‐free sensing through a gating effect on conductance upon the target binding to the receptor so generating a measurable output. Gating by the target or analyte can be achieved through various mechanisms including changes in local carrier density and changes in local electrostatics or direct charge input. FET‐based sensors typically employ metal‐oxide‐semiconductors (MOS) or organic semiconductors as channel materials, as they are cost‐effective, scalable, and have system‐on‐chip (SoC) capabilities. However, MOSFETs also have limitations such as poor signal‐to‐noise ratio, low on/off ratio, slow mobility, and a short lifetime.[^19^, ^20^, ^21^] To overcome these limitations, carbon nanomaterials have been proposed as alternative channel materials for FET devices due to their desirable chemical and physical properties, including: ease and breadth of chemical functionalisation strategies; (size) compatibility with biomolecules; their excellent electronic and mechanical properties. These chemical and physical characteristics of carbon nanomaterials can assist in the sensitive detection of target biomarkers with FET biosensing devices setups. For example, the variety of chemical functionalisation strategies can allow an optimised control over the site‐specific attachment of the biomolecule of interest and the minimisation of non‐specific adsorption. The nanoscale size (comparable to biomolecules) can result in lower test sample volumes, while the excellent electronic properties of carbon nanomaterials can potentially increase the signal‐to‐noise ratio of the devices. Table 1 summarises FET biosensor developments in terms of materials employed and their limit of detection.

**Table 1:** Comparison of existing FET biosensors in terms of materials, FET structure, and limit of detection.

	Materials	FET structure	Analyte	Receptor	Detection method	Limit of detection
**Nanocarbons**	swCNT^[22]^	Bottom gate	DNA	Complementary DNA	I_ds_−V_g_	0.87 aM
	swCNT^[23]^	Liquid gate	Protein	Peptide aptamer	I_ds_−V_g_	2.3 pM
	swCNT^[24]^	Bottom gate	Chemical	Protein	I_ds_‐Time	1.2 nM
	Graphene oxide^[25]^	Top gate	Protein	DNA aptamer	I_ds_−V_g_	0.23 pM
	Graphene^[26]^	Bottom gate	Exosome	Antibody	I_ds_−V_g_	0.1 μg/mL
	Graphene^[27]^	Bottom gate	Protein	Protein	I_ds_−V_g_	0.37 pM
	Graphene^[28]^	Side gate	Protein	DNA aptamer	I_ds_−V_g_	2.6 pM
	Graphene^[29]^	Liquid gate	Chemical	DNA aptamer	I_ds_−V_g_	0.01 nM

**Metal**	In_2_O_3_ ^[30]^	Side gate	Chemical	Enzyme	I_ds_−V_g_	10 nM
	Si nanowire^[31]^	Liquid gate	Ion	Chemical receptor	I_ds_−V_g_	10 μM
	ITO nanowire^[32]^	Bottom gate	DNA	Complementary DNA	I_ds_−V_g_	1 fM
	MoS_2_ ^[33]^	Bottom gate	Protein	Antibody	I_ds_−V_g_	1 ng/L
	WS_2_ ^[34]^	Bottom gate	DNA	Complementary DNA	I_ds_−V_g_	3 aM

**Organic semiconductor**	Pentacene^[35]^	Extended gate	Protein	Antibody	I_ds_−V_g_	1 ng/mL
	Polyethylene naphthalate^[36]^	Extended gate	Protein	Chemical receptor	I_ds_−V_g_	0.6 pM
	PDPP3T^[37]^	Bottom gate	Chemical	Protein	I_ds_−V_g_	0.1 nM

**Figure 2:**
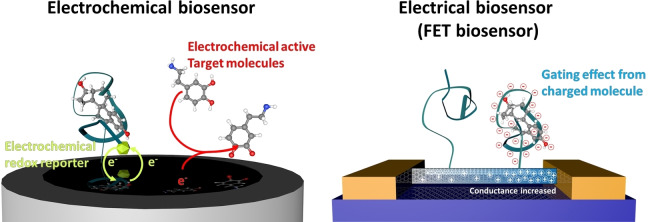
Comparison of detection mechanisms used in an electrochemical biosensors and FET electrical biosensors.

FET‐based electrical biosensors may also be compared with electrochemical biosensors, due to their similarity in size, ease of use and ability to use a variety of different receptors to target different analytes. However, key differences lie in the sensing mechanism. Electrochemical biosensors rely upon a redox reaction between an electrochemically active species and the electrodes to produce a change in current flow (Figure 2). If the target analyte itself is not electrochemically active, an electrochemical redox reporter must be used as the receptor; this limits electrochemical sensing to a few selected systems (e. g., protein sensing using electrochemical redox reporter tagged aptamer,^[38]^ and enzyme activity sensing using probe peptide modified electrode^[39]^). FET‐based electrical biosensors, on the other hand, do not rely on signal generators and can monitor binding events between potentially any target and receptor pair through the gating mechanisms mentioned above. Table 2 shows the different biosensing mechanism of electrochemical biosensors compared to FET‐based electrical biosensors.

**Table 2:** Comparison of sensing performances between FET‐based electrical biosensors and electrochemical biosensors.

Analyte	Receptor	Materials	Sensing platform	Sensing technique	Limit of detection
**L–Cysteine**	Chemical receptor	swCNT^[40]^	FET	I_ds_‐Time	0.45 fM
	(none)	CuFe_2_O_4_/reduced graphene oxide^[41]^	Electrochemistry	Cyclic voltammetry	0.383 μM

**Amyloid‐β**	DNA aptamer	swCNT^[42]^	FET	I_ds_−V_g_	1 fM
	Peptide receptor	Au‐Ppy‐3‐COOH^[43]^	Electrochemistry	Electrochemical impedance spectroscopy	1 nM

**Protein kinase A**	Peptide receptor	swCNT^[44]^	FET	I_ds_‐Time	1.2 mU/mL
	Peptide receptor	AuNP/reduced graphene oxide^[45]^	Electrochemistry	Electrochemical impedance spectroscopy	53 mU/mL

**Glutamate**	Enzyme	swCNT^[46]^	FET	I_ds_‐Time	92 pM
	Enzyme	PtDENs/swCNT^[47]^	Electrochemistry	Amperometric i‐t curve	6.64 μM

### 1.3 The importance of NC‐FETs to the field of biosensing

The development of NC‐FET biosensors has had a major impact on both electronics and biology. With respect to electronics, the major development centres around constructing devices with single CNTs or graphene sheets. These nanoscale electronic device setups overcome issues regarding the macroscale size, overheating, and energy consumption.[^48^, ^49^, ^50^, ^51^] However, devices fabricated with single‐nanomaterial resolution currently suffer from manufacturing scalability issues.

Be it environmental or healthcare applications, there is a drive to move away from centralised lab facilities to point of use testing. For example, personalised home testing, including real‐time disease monitoring, could revolutionise healthcare.[^52^, ^53^, ^54^, ^55^] The development of NC‐FET biosensors will ultimately have a significant impact on the field of biosensing for the reasons outlined below. Firstly, there will be no need for complex sample preparation prior to testing. This means the target can be measured in its “native” environment and state. NC‐FETs can be flexible in terms of what they can sense. By changing the receptor, different target analytes can be detected as the underlying gating mechanisms will be the same. The devices will be small, as NC‐FETs have simple structures on the millimetre‐scale comprising two electrodes and one semiconducting channel. This in turn opens up the possibility of NC‐FETs being incorporated into wearable and implanted systems.

### 1.4 Current limitations of NC‐FET

While there is great promise for NC‐FETs as biosensors, some issues still need to be resolved for successful long‐term applications and commercialisation. These include lack of uniformity in nanocarbon deposition when fabricating the devices on a large scale, complex preparation protocols for nanocarbons (especially single tube/sheet channels), and non‐specific analyte binding events.[^56^, ^57^, ^58^, ^59^] To address uniformity in fabrication, the immobilisation (or transfer) of nanocarbons to substrate can be optimised by changing the immobilisation parameters, including concentration, time, temperature, and solvent.[^60^, ^61^, ^62^, ^63^, ^64^, ^65^] Improvements in synthesis, purification, and separation of nanocarbon have simplified the fabrication of NC‐FETs.[^66^, ^67^] Non‐specific binding events can be prevented by passivation using polymers or chemicals such as polyethylene glycols (PEG), Nafion^TM^, surfactants, and bovine serum albumin (BSA).[^68^, ^69^, ^70^, ^71^, ^72^, ^73^, ^74^, ^75^, ^76^] However, these surface modifications can reduce sensitivity by providing a barrier that masks the incoming analyte from the nanocarbon. Thus, approaches such as designed interfacing of high affinity receptors (see below) or the use of charged species such as DNA will also help.

### 1.5 Focus of this review

Previously, Kong,^[77]^ Yum,^[78]^ Khosla,^[79]^ and Star *et al*.,^[80]^ published review papers about the application of nanoscale carbon in chemical (bio)sensors, electrochemical (bio)sensors, and electrical (bio)sensors. These reviews present the preparation of materials, the properties investigation, and the popular applications of nanoscale carbons. In this review, we focused on providing a fundamental basis for FET‐based biosensors in the view of both physics and biology.[^77^, ^78^, ^79^, ^80^] Furthermore, this review provides an overview of the most recent advancements in the fabrication of NC‐FET biosensors, and the functionalisation techniques central to their biosensing capabilities. We first introduce the fundamental basis by which NC‐FETs operate, and how this can be exploited as a biosensing electrical platform. We then discuss the synthesis and assembly of nanocarbon that acts as the basal conductor in NC‐FET devices. We next outline the different strategies employed for the functionalisation of nanocarbon with biomolecules, and the alternative approach of functionalising biomolecules for nanocarbon attachment. Specific examples will be given to exemplify the range and success of different functionalisation techniques. Finally, we will offer some future perspectives on NC‐FET development; where research is heading and how we can improve NC‐FET design.

## 2 Focus on the Operation Mechanisms of FET Biosensors

To accurately measure signals from electrical biosensor devices and to design optimal FET biosensors, it is necessary to understand their operational mechanism.

### 2.1 Structure of FET devices

**Figure 3:**
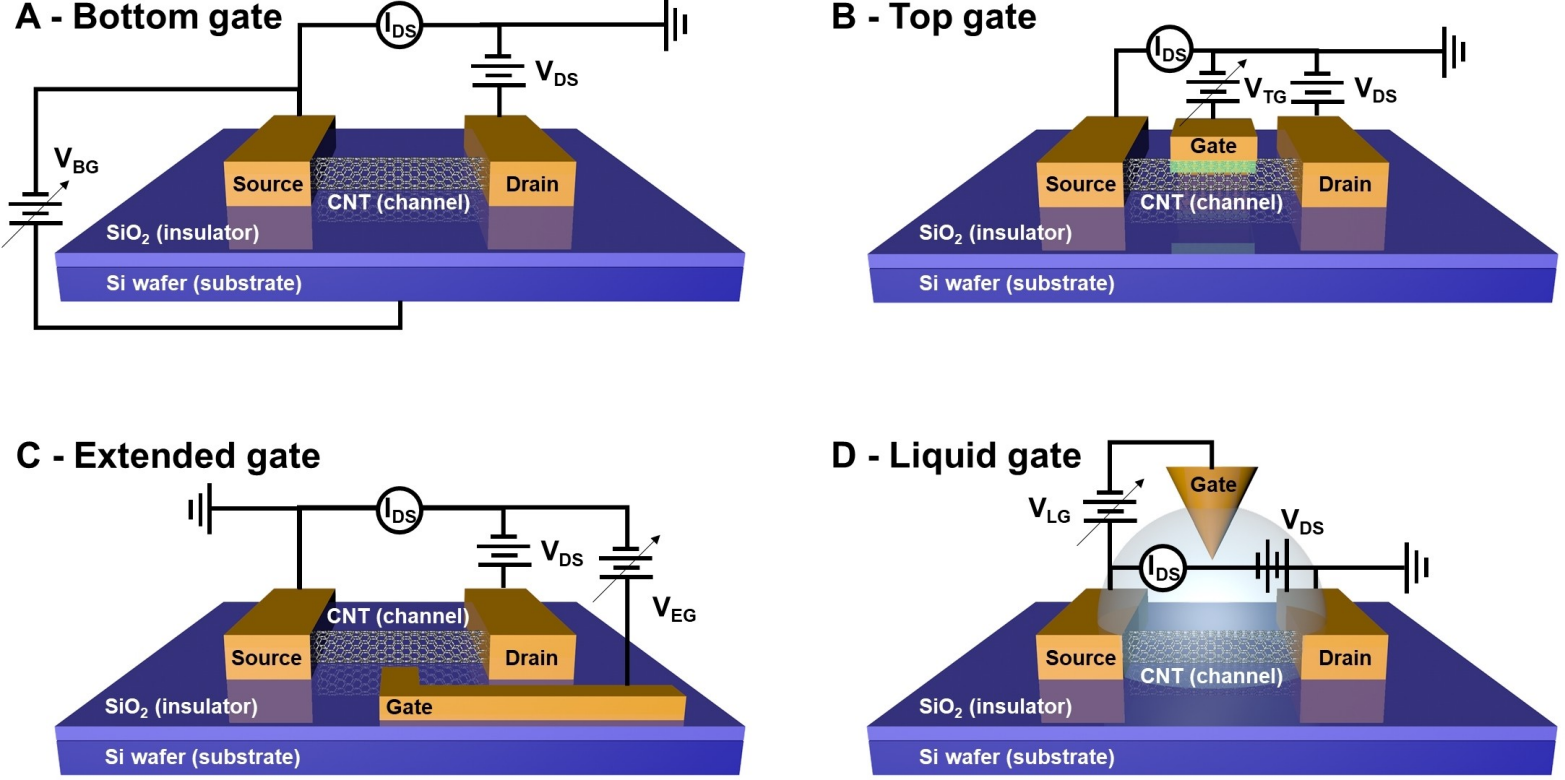
Structures of different types of FET devices.

FET devices are composed of three key parts: the channel, the electrodes comprising the source (S), drain (D) and gate (G), and the base substrate. The channel is formed by connecting the S and D electrodes using a semiconducting material. For the purposes of this review, we will focus only on nanocarbon as the channel material, but others include metal‐oxide semiconductors, organic semiconductors, and silicon nanowire. The gate electrode regulates the current flowing between the S and D electrodes, and four different types exist: bottom, top, extended, and liquid (Figure 3). The gate is chosen based on the purpose of the experiment. The bottom gate is the most common type of FET structure and can allow the exploration of general electrical characteristics for biosensing, such as threshold voltage shift, conductance changes, and on‐off ratio changes. The top and extended gates are appropriate for studying charged analyte gating effects without damaging channel materials. Alternatively, with a liquid gate it is possible to focus on a narrow gate potential range, which can in turn allow users to correlate detailed gating effects to biosensing events.

Finally, the base substrate uses a dielectric insulating material, most commonly silicon dioxide, but glass, polydimethylsiloxane (PDMS), and polyethylene terephthalate (PET) are also used.[^81^, ^82^, ^83^, ^84^] The electrical signals of FET devices are typically measured with a semiconductor parameter analyser (or source meter) by applying voltages between the source and gate electrodes.

### 2.2 Measuring changes in conductance and threshold voltages

The current (or conductance) between the source and drain (I_SD_) depends on the source‐drain voltage (V_SD_) and/or gate voltage (V_G_). Typically, the I_SD_ increases with increasing V_SD_, in an ohmic contact characteristic. When a p‐type semiconductor channel material is used, where the main charge carrier is represented by positively charged holes rather than negatively charged electrons, the I_SD_ is drastically decreased upon sweeping V_G_ from negative to positive values; the turn‐off point is called the threshold voltage. The largest change in current is observed at the threshold voltage, and hence this value is taken forward and applied to FET devices for analyte sensing experiments.

**Figure 4:**
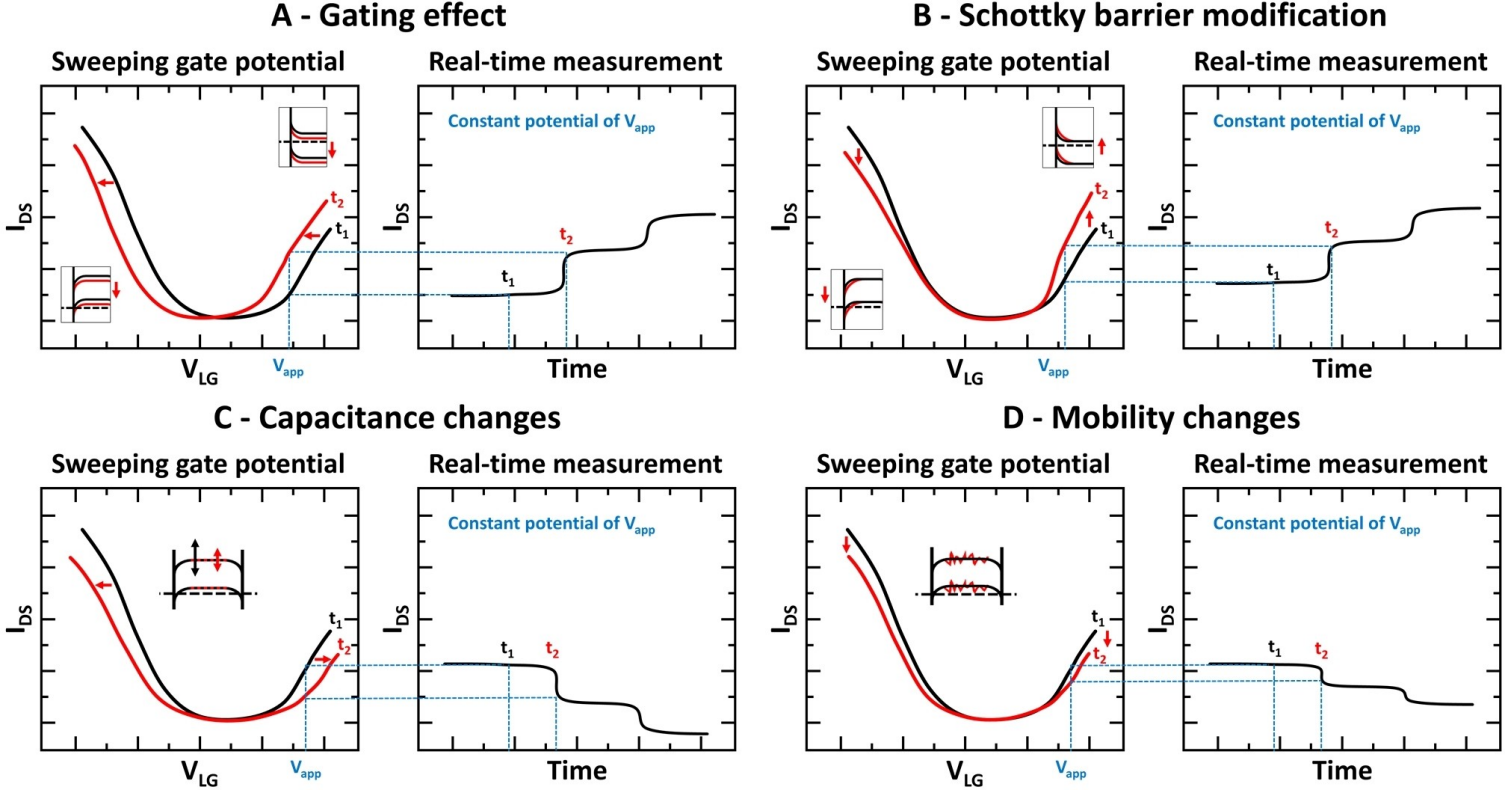
Mechanisms of detecting analyte binding through changes in FET transfer characteristics. I_DS_ is source‐drain current, V_LG_ is applied potential to liquid gate, and V_app_ is applied potential to gate with constant value for real‐time sensing. Insets represent the energy bands of the channels.

Two main methods can be used to relate output signals in FET devices to analyte binding: i) measuring the conductance of source‐drain electrode, and ii) measuring the shift of the threshold voltage. Measuring current (or conductance) of the source‐drain electrodes can be performed in real‐time, where I_SD_ is monitored before and after the addition of the target analyte, with an applied constant potential of V_SD_ and V_G_ during the measurements. To observe a shift in the threshold voltage, I_SD_ is measured while V_G_ is swept (at constant V_SD_). Shifts in output signal can be induced by different, and in some cases, competing effects such as outlined in Figure 4.: i) Gating resulting in a shift in FET transfer characteristics; ii) Schottky barrier modifications induced by molecular adsorption (changes in the local work function and band alignment of the channel material);[^85^, ^86^] iii) Capacitance changes, where the reduction in gate efficiency may occur due to the low permittivity of the adsorbed biomolecules relative to the electrolyte, which limits the gate capacitance; iv) Changes in mobility, e. g. the slope of the FET transfer characteristics can be reduced by changes in charge mobility which gives rise to a decrease in the conductance without shifting the FET transfer characteristics to gate voltages.^[87]^

### 2.3 Gating via analyte charge

**Figure 5:**
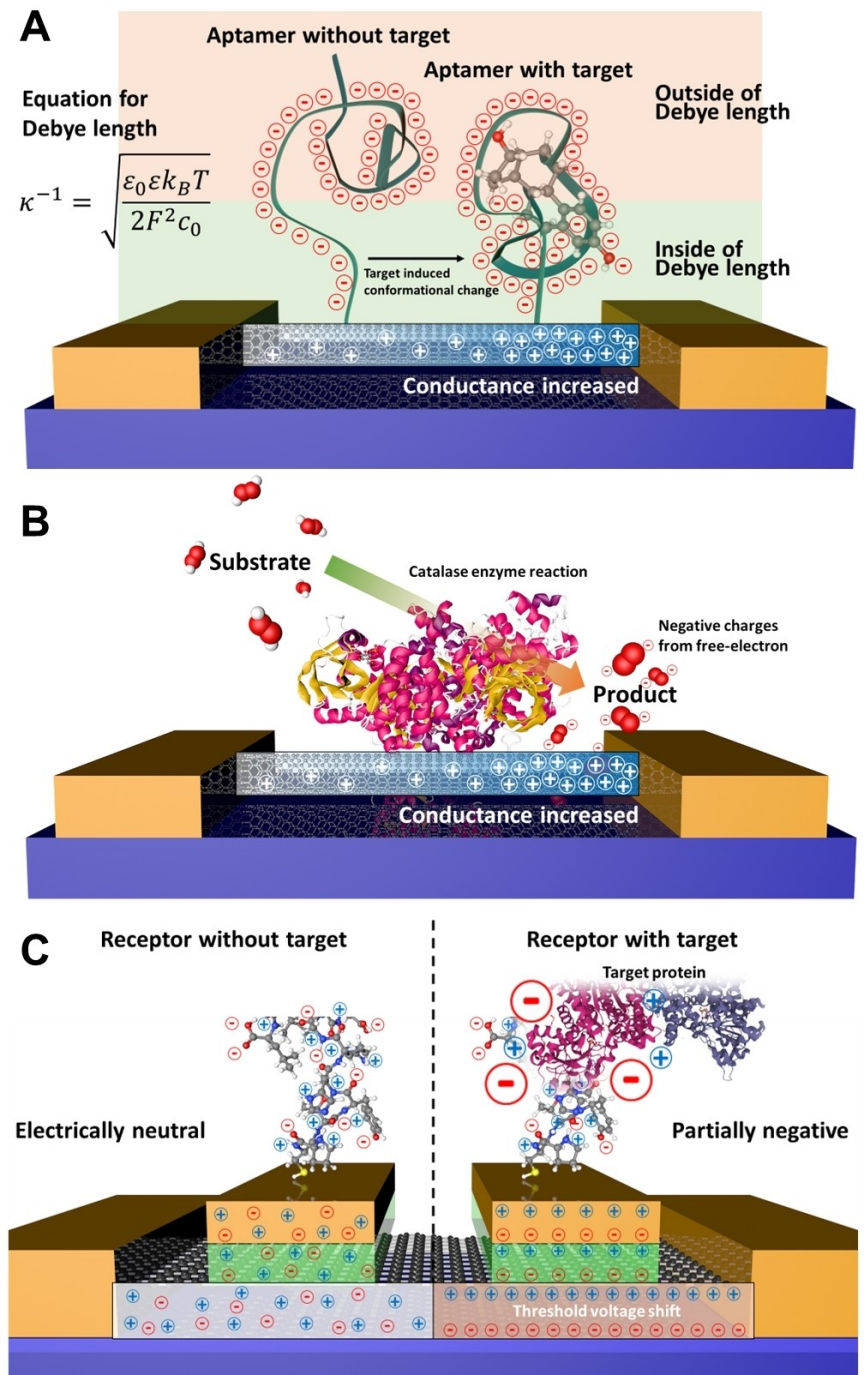
Signal modulation pathways for receptors immobilised on FET devices. Signals can be generated by: A. conformational changes of the charged receptors changing the charge within the Debye length; B. charged molecules from an enzymatic reaction; and C. changes in local charge upon analyte binding.

For biosensing using NC‐FETs to be possible, binding of the analyte to the nanocarbon‐bound receptor must be transduced into an electrical signal (Figure 5).[^5^, ^6^, ^7^] The conductance/resistance changes in FET biosensors relies on the gating effect by charged molecules located within the Debye length. The equation below allows the calculation of the Debye length ($\lambda_{D}$ ) in aqueous phase at room temperature:

$$\lambda_{D}=\frac{1}{\sqrt{4\pi l_{B}\sum_{i}\rho_{i}z_{i}^{2}}}$$

where $l_{B}$ is the Bjerrum length (0.7 nm), and $\rho_{i}$ and $z_{i}$ are the density and valence, respectively, of ionic species.^[88]^ When charged molecules are attached to FET devices, the surface potential of the channel is modulated. These changes in the Debye length cause accumulation or depletion of carriers in the FET channel, thus increasing or decreasing conductance. This is known as the charged molecule gating effect.^[89]^

Three different kind of target analyte molecules can typically be considered: charged small molecule, small molecules with no charge, and large macromolecules. When small charged molecules bind to FET devices, changes in conductance can be caused by the charged molecule gating effect. If the small molecule has no charge, gating needs to induced through changes in the receptor. For example, binding of the uncharged analyte to a DNA aptamer or protein induces a conformational change in receptor resulting in a change in the net charge within the Debye length. This in turn induce a change in the FET biosensor conductance/resistance.^[58]^ The situation is more complex for biomacromolecules and generally is dependent on the change in charge within the Debye length. For example, proteins have complex electrostatic surfaces comprised of negatively and positively charged patches. The gating effect will depend on the nature of the electrostatic surface that comes with the Debye length. This opens up the potential to use the unique electrostatic properties of an individual protein to act as its electrical signature. For nucleic acid‐based aptamer receptors, the main driver is change in the negative charge population (derived from the phosphate backbone) within the Debye length on analyte binding. In each of the above case, FET‐based detection is dependent on the Debye screening effect.

The Debye screening effect is caused by the solvation of ions (or charged small molecules) dispersed in an ionic solution, which lowers the effective charge of the incoming macromolecular analyte:[^91^, ^92^, ^93^, ^94^] ions or charged small molecules form an electric double layers. If solvent ionic conditions are too high (e. g., high salt) then the Debye length will be very short. For example, the Debye length for phosphate buffered saline (PBS), a common buffer used to mimic physiological conditions, has a calculated Debye length of 0.7 nm. This in essence means that any analyte will change the charge environment within 0.7 nm of the nanocarbon channel. Thus, it is important to consider both the buffering conditions (e. g., remove NaCl from PBS) and/or how the receptor is bound to the nanocarbon. Many studies proposed different approaches to overcome the Debye screening effect, including the use of tailored DNA aptamers, morphology improvement, the use of enzymatic reactions, modifying polymers, and device modulation.[^95^, ^96^, ^97^, ^98^] Nonetheless, the Debye screening effect is still one of the most critical drawbacks for the commercialisation of NC‐FETs.

## 3 Preparation of NC‐FETs: from Synthesis of Nanocarbons to Device Fabrication

### 3.1 Carbon nanotubes: Synthesis and preparation

In 1991 Iijima first described the synthesis of carbon nanotubes using the arc discharge evaporation method.^[99]^ Since then, carbon nanotube synthesis methods have developed rapidly to include chemical vapor deposition,^[100]^ vapor‐phase growth,^[101]^ plasma enhanced chemical vapor deposition,^[102]^ and flame synthesis.^[103]^

The biggest hurdles for CNTs are related to the purification and separation of the nanotubes due to the presence of amorphous carbon and metal nanoparticles. Furthermore, CNTs have various chiral forms that exhibit different physical characteristics. Therefore, various purification techniques have been developed, such as chemical oxidation, filtration, sonication, and chromatography.[^66^, ^67^, ^104^] These techniques use differences in the chemical and physical characteristics of CNTs including radius, length, chemical resistance, structural stability, and solubility.[^61^, ^105^] For commercial applications scalable purification and separation of CNTs is still needed, but it is clear from smaller scale studies that this is feasible.

### 3.2 Carbon nanotubes: Device fabrication and electrical features

When fabricating NC‐FETs with CNTs, two approaches can be used. One is patterning the electrodes onto the CNTs previously deposited/grown on surfaces, and the other is depositing CNTs from solution onto pre‐patterned electrodes.

Immobilising CNTs onto pre‐patterned electrodes is far more amenable to large scale fabrication of NC‐FETs.[^106^, ^107^, ^108^] There are a number methods to immobilise CNTs including drop‐casting, dip‐coating, and dielectrophoresis (DEP).[^109^, ^110^, ^111^, ^112^, ^113^]

Drop‐casting involves placing CNT solutions onto pre‐patterned electrode chips and allowing the solvent to evaporate. The CNTs are immobilised by physisorption.[^114^, ^115^]

In Dip‐coating, the pre‐patterned electrode chips are dipped into a CNT solution with immobilisation occurring across channel through van der Waals forces.[^46^, ^116^, ^117^, ^118^] In DEP CNTs are immobilised by applying an AC bias between electrodes that drives solution‐cast CNTs along the electric field gradient.[^113^, ^119^, ^120^, ^121^]

### 3.3 Graphene: Synthesis and preparation

Graphene is a commonly used 2D nanomaterial for electronic devices due to its excellent electronic properties including ballistic electron transportation, high thermal conductivity, and mechanical flexibility.[^122^, ^123^, ^124^, ^125^, ^126^] Approaches to its synthesis can be classified as ‘bottom‐up’ or ‘top‐down’. Chemical vapor deposition (CVD) is among the most frequently used ‘bottom‐up’ methods for synthesizing graphene due to its simple procedure and high‐quality end product.[^127^, ^128^, ^129^] However, large scale synthesis by CVD is challenging so potentially of limited scope for commercial applications.

‘Top‐down’ approaches for synthesizing graphene include mechanical exfoliation, liquid‐phase exfoliation, and chemical reduction of graphene oxide. These are easier to implement for large‐scale synthesis and allow for facile functionalisation, but there is less control over the 2D‐material's size (of the flakes/monolayers) and quality, from defects in nanomaterial homogeneity.[^130^, ^131^]

### 3.4 Graphene: Device fabrication and electrical features

To fabricate graphene FETs, synthesised graphene must be transferred to the substrate (glass, polymer, silicon wafer, etc.). Typically, CVD‐synthesised graphene is transferred from a copper substrate to the target substrate by using a stamping method.[^132^, ^133^] Solution‐synthesised graphene is usually drop‐cast onto the substrate; subsequently electrodes are patterned by metal evaporation.

**Figure 6:**
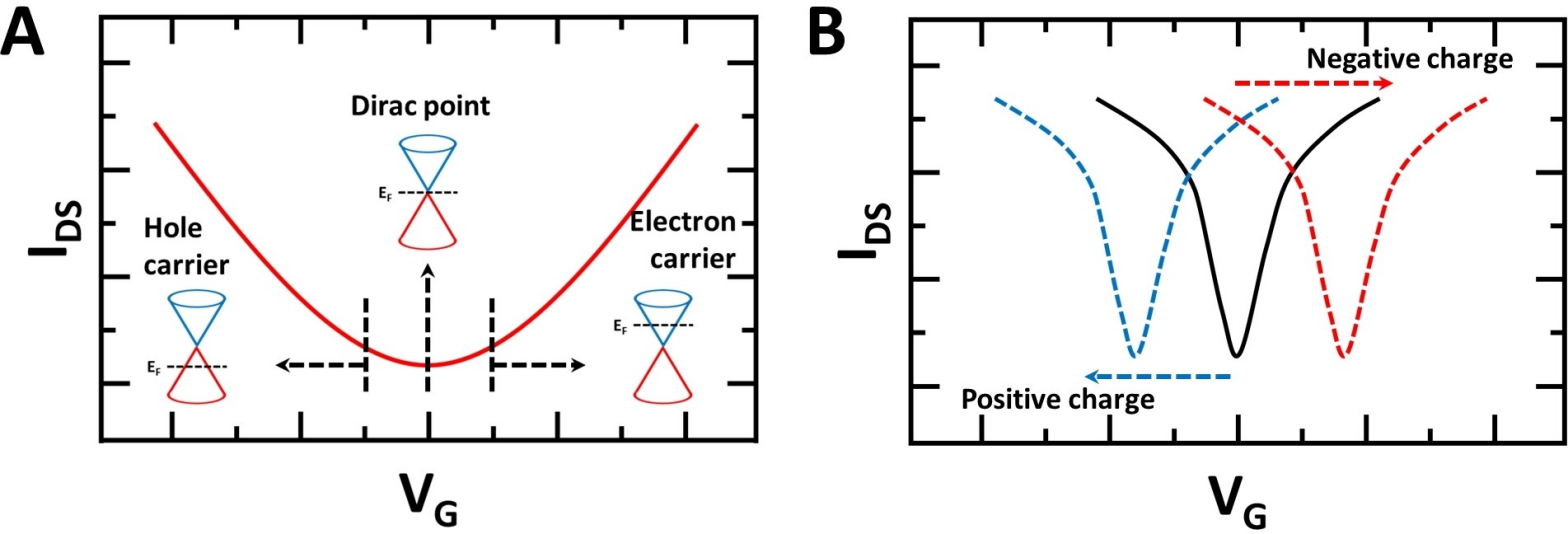
A. FET transfer characteristics with the band structures for majority of electron transfer at each regime. B. Changes in transfer characteristic by charged molecular gating via charged analyte.

Typically, graphene FETs exhibit a V‐shaped curve for I_DS_ upon sweeping the gate voltage from negative to positive.^[134]^ This indicates the polarity of the majority charge carriers changes in the graphene layer: the negative potential indicates an increasing density of hole carriers, while the positive potential indicates a rising density of electron carriers.[^135^, ^136^, ^137^] As shown in Figure 6A, the valley of the V‐shaped I_DS_ curve shows the minimum value of the density of charge carriers, referred to as the Dirac point. Moreover, a left‐shifted neutrality point indicates an effect due to a positively charged analyte, and a right‐shift from negatively charged analyte (Figure 6B).[^138^, ^139^, ^140^, ^141^, ^142^]

## 4 Functionalisation of Nanocarbon for Biosensing Applications

**Figure 7:**
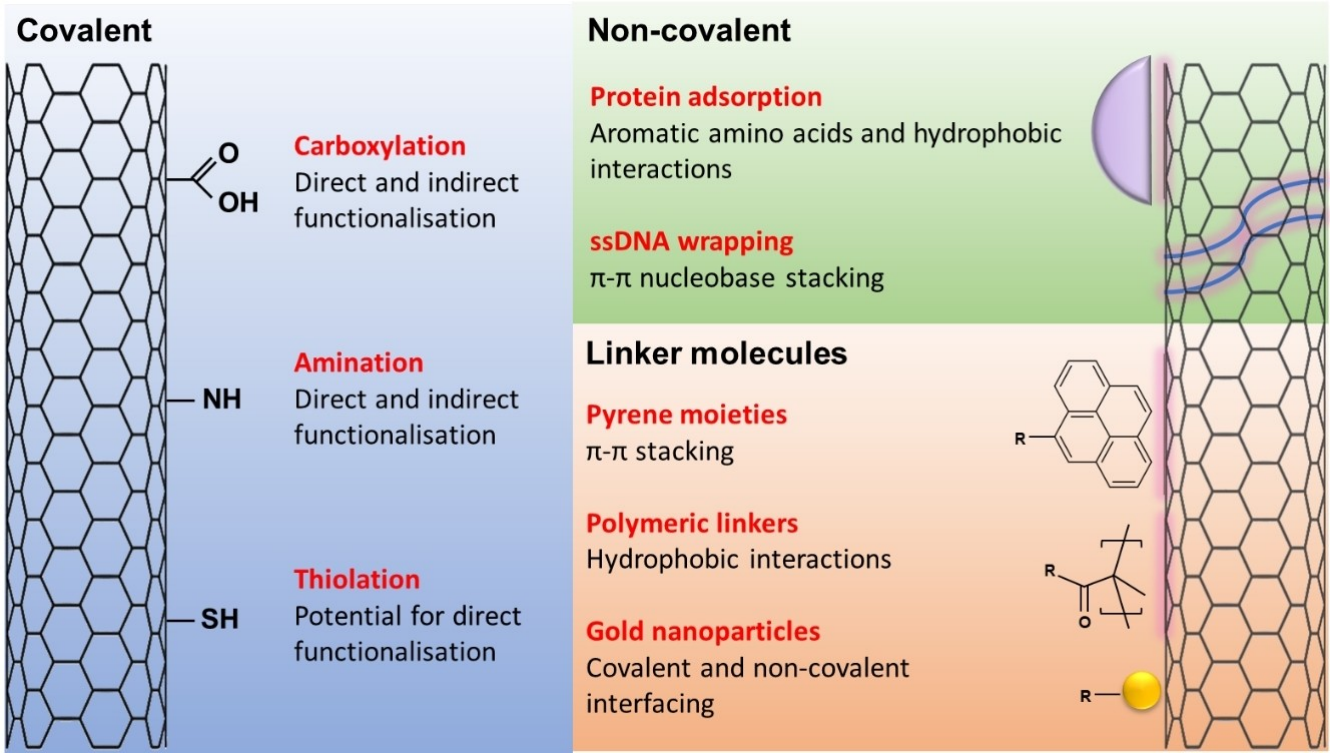
Biocompatible nanocarbon functionalisation strategies. Covalent modification introduces functional groups directly into the carbon lattice, and these can conjugate biomolecules directly or indirectly (via attachment of linker molecule). Non‐covalent functionalisation utilises hydrophobic interactions and π–π stacking of biomolecules to directly decorate the surface of nanocarbon. Linker molecules can play an adaptor role; covalently or non‐covalently interfacing with nanocarbon, whilst providing reactive biochemical handles for biomolecule conjugation.

Once the basic NC‐FET has been set up, nanocarbon materials can undergo functionalisation to introduce biochemically compatible handles and linker groups. This can be done via covalent or non‐covalent approaches (Figure 7) and allows the selective and controllable interfacing with the required receptors for target analytes. Both strategies offer distinct advantages and disadvantages. For the purpose of this section, carbon nanotubes and graphene will be collectively described as ‘nanocarbon’ due to the similar methodologies in place used to functionalise these materials, and specific application examples will be mentioned for both.

### 4.1 Covalent functionalisation

Covalent functionalisation of nanocarbon can be defined as the breaking of the sp2 carbon lattice to create a new chemical bond.^[143]^ This represents a vast field of research,[^144^, ^145^, ^146^, ^147^] with reviews by Guy and Walker^[147]^ and Dubey *et al*.,^[148]^ going into great detail on the wet and dry chemistry techniques behind these modifications. Whilst electron mobility may be slightly hampered,^[149]^ the flexibility and functionality of new chemical groups increases nanocarbon usefulness as a transistor. In particular, it offers NC‐FETs discrete sites for direct and indirect biomolecule conjugation (Figure 7) so creating a spatially intimate environment for binding events to be monitored.^[150]^ For these reasons, covalent functionalisation remains a popular strategy in NC‐FET design, and a range of examples will be discussed below.

Carboxylation is one of the most common functionalisation techniques,^[143]^ as carboxyl groups make a prime target for further derivatisation. Oxygen‐containing groups are typically introduced by a strong oxidising agent[^144^, ^147^, ^151^] with oxidised graphene undergoing a further reduction process to establish semi‐conducting behaviour.[^152^, ^153^] Biomolecule conjugation can then take place directly or indirectly. EDC/NHS (1‐ethyl‐3‐(3‐dimethylaminopropyl)carbodiimide/*N*‐hydroxysuccinimide) coupling is a popular choice for direct protein attachment[^154^, ^155^] with stable amide bonds formed between the free amines in the peptide backbone or side chain, and the activated NHS‐ester.^[156]^ Indeed, this was the functionalisation method of choice in the recently developed SARS‐CoV‐2 antigen FET‐biosensor. Polyclonal antibodies to the spike 1 protein were conjugated onto SWCNTs to achieve ultrasensitive levels of antigen detection (0.55 fg/mL).^[157]^ This illustrates the potential NC‐FETs have to modernise diagnostic techniques, replacing time consuming laboratory‐based assays. Carboxyl groups can further be used as sites of linker molecule attachment,^[148]^ but in the ever‐evolving world of NC‐FETs, this is perhaps not as desirable; combining the reduced conductivity of the defected sp2 lattice with a linker that may push the conjugated biomolecule beyond the Debye length.

Amination is a less commonly employed functionalisation technique, but like carboxylation, it has the potential to react with native functional groups in biomolecules. Primary amines are typically introduced through substitution of oxygen‐containing groups on graphene oxide,[^158^, ^159^, ^160^] but exposure of pristine graphene to Ar/NH_3_ plasma has also been shown to work effectively.^[161]^ Direct conjugation with proteins can then take place via carbodiimide activation of native carboxylic acid groups; a process observed in Islam *et al*.’s graphene FETs, which used conjugated antibodies to detect biomarkers associated with HIV, cardiovascular disorders and rheumatoid arthritis.^[162]^ Indirect conjugation to ssDNA via glutaraldehyde was also used by Baraket *et al*. as a first proof of concept for DNA detection by this method.^[161]^

Thiolation is comparatively new to the field of carbon functionalisation but holds great promise in the development of NC‐FETs. Thiol groups can specifically target cysteine residues in proteins to form disulphide bonds. Surface exposed cysteines with free thiol groups are relatively rare in proteins (compared to, for example, lysine) so can offer more control over protein orientation when it binds the nanocarbon. This would ensure the active site of an enzyme, or the antigen‐binding site of an antibody, would protrude away from the nanocarbon surface and allow interaction with the analyte in question. Furthermore, the chemical simplicity of a disulphide bond means it needs only reducing conditions, or exposure to a reductase, to reversibly remove the receptor protein, ‘resetting’ the biosensor for further experiments. Whilst this approach has thus far been limited to gold substrates,[^132^, ^133^] developments in the fabrication of thiol‐functionalised CNTs by a two‐step process of polysulfide diradical addition and sodium borohydride reductive cleavage,^[165]^ offers an exciting new template in future NC‐FET design.

### 4.2 Non‐covalent functionalisation

Non‐covalent functionalisation is the alternative strategy to covalent approaches, for interfacing nanocarbon with biomolecules. Hydrophobic interactions, electrostatic interactions or π–π stacking between the two species provide bioreactivity without compromising the structural or electrical integrity of nanocarbon.^[166]^ Whilst this has obvious advantages over covalent functionalisation, it is important to consider the factor of Debye length in the NC‐FET design. Proteins, such as antibodies, are large in size, so the use of physiologically relevant high salt buffers can place the functional centres of these proteins beyond the sensing region of the biosensor.^[150]^ The design, especially in terms of the interface site between the receptor and nanocarbon, is therefore crucial to the success of non‐covalently functionalised NC‐FET.

Protein adsorption is the simplest method to directly functionalise nanocarbon, using π–π stacking from aromatic amino acids and general hydrophobic interactions from the protein surface. Some of the earliest attempts at interfacing proteins with nanocarbon used the adsorption of enzymes, such as glucose oxidase^[167]^ and α‐chymotrypsin.^[168]^ Whilst the former enzyme displayed evidence of electron transfer, the latter retained only 1 % of its activity due to it unfolding on SWCNT. Weak physical bonding between the protein and nanocarbon also contributes to a loss of protein immobilisation,^[166]^ consequently meaning this strategy tends to be avoided in the design of modern NC‐FETs. However, the importance of these early experiments will not be forgotten, as they highlight the importance of protein structure and function in these hybrid systems.

In contrast to proteins, ssDNA represents a biomolecule that is highly suited to non‐covalent interaction with nanocarbon. The inherent structure of the polymeric nucleic acid, in particular, complements SWCNTs; with planar nucleobases forming π–π stacking interactions with the sidewalls, and the polar sugar‐phosphate backbone providing sufficient torsion to wrap the nanotube.^[169]^ The natural amphiphilic structure of ssDNA means it is also often employed in the preliminary stages of SWCNT functionalisation, to aid dispersion and control the available surface area for protein functionalisation.[^170^, ^171^]

In terms of its applications, ssDNA use in NC‐FET design is expansive, being used directly as a DNA probe to sense for target sequences and also indirectly, with additional chemistries being incorporated to create a platform for further biomolecule attachment.[^171^, ^172^] For example, research by Xu *et al*. used ssDNA containing bicyclononyne to wrap around SWCNTs, allowing azide‐functionalised aptamers towards a cortisol‐biomarker to bind in a cycloaddition reaction.^[113]^ A consideration for the use of ssDNA, however, comes from Moon *et al*., who demonstrated that nucleases present in cells are sufficient to degrade ssDNA wrapped around SWCNTs.^[173]^ With a substantial number of NC‐FETs designed to be testing medical samples, preparations need to be in place to prevent the hydrolytic enzymes from destroying the biosensor upon sample deposition.

### 4.3 Linker‐mediated functionalisation

Linker molecules represent the final category of functionalisation and can be characterised through their bifunctional ability to covalently or non‐covalently interact with nanocarbon, whilst simultaneously providing a reactive handle to covalently interface with a biomolecule. Coming under the same wing as non‐covalent functionalisation, this approach again benefits from the undisrupted sp2 network; but is at risk of pushing biomolecules beyond the Debye length.[^146^, ^174^]

Pyrene based linkers represent a significant proportion of these linkers, utilizing their aromatic pyrene group to π‐stack onto nanocarbon.[^40^, ^175^] Commonly employed across graphene and SWCNTs, is 1‐pyrenebutanoic acid succinimidyl ester (PBASE), which uses the ester group in a nucleophilic substitution reaction with free amines on a protein, anchoring the protein to the pyrene and thus the nanocarbon.[^140^, ^141^, ^142^, ^176^, ^177^] Indeed, another SARS‐CoV‐2 NC‐FET was developed using this method, anchoring the spike protein antibody onto PBASE coated graphene.^[178]^ The sensitivity of this biosensor reached levels of 1 fg/mL in phosphate buffered saline, but the Debye effects became apparent once samples were tested in clinical transport medium, where sensitivity reduced 100‐fold. Another pyrene‐based approach uses a dibenzylcyclooctyne (DBCO) group instead of an ester. This uses the click chemistry reaction of strain‐promoted azide‐alkyne cycloaddition when reacted with proteins containing an azide group. Xu *et al*., made an important development by incorporating these azide mutations at specific residues, allowing the receptor protein to be conjugated to nanocarbon in different, defined orientations, which resulted in differential gating effects being observed.^[174]^

Polymeric linkers are amphiphilic molecules typically consisting of a hydrophobic alkyl backbone with protruding polar groups for biofunctionalisation and water solubilisation.^[179]^ Acting in much the same way as pyrene‐based linkers, polymeric linkers are larger in size and can wrap helically around carbon nanotubes,^[148]^ whilst films are formed on graphene.^[180]^ Polymers devised by Martínez *et al*. were used to coat SWCNTs, trapping electrons and holes upon DNA hybridisation and allowing a measured response.^[181]^ The polymer contained heterofunctional groups, with *N*‐succinimidyl ester covalently binding the aminated ssDNA probes, and polyethylene glycol and methacrylate used to prevent the non‐specific adsorption of protein. This demonstrates the flexibility polymeric linkers can provide in NC‐FET design.

Gold nanoparticles (NPs) are a final example of linker molecules commonly used in NC‐FET design. These are a favoured linker because of their biocompatibility, with a naturally high affinity to thiol groups^[182]^ and easily modifiable surface, to conjugate biomolecules via EDC/NHS chemistry.^[183]^ The incorporation of gold NPs within the nanocarbon network can then take place covalently via a variety of deposition techniques[^184^, ^185^] or bind non‐covalently via ssDNA^[186]^ and pyrene linkers.^[187]^ This has subsequently led to the development of many novel NC‐FET devices, including the first protein‐based photosensing system on graphene, where photosystem I was immobilised to gold NPs via EDC/NHS chemistry, and the NP itself was non‐covalently attached to the graphene via a pyrene‐based linker. This setup saw an increase in conductivity after red‐light irradiation; a steppingstone in the fabrication of protein‐based light sensors and solar cells.^[188]^ Another impressive development was the graphene‐based FET by Danielson *et al*., which used gas‐phase synthesised gold NPs to anchor thiol‐modified DNA aptamers. These aptamers were designed to sense streptavidin and hybridise with cDNA, reaching attomolar (10^−18^) detection levels and discriminating between sequences containing single nucleotide polymorphisms.^[185]^ Gold NPs will thus undoubtedly continue to feature in future NC‐FET design.

## 5 Functionalisation of Biomolecules for Nanocarbon Attachment

**Figure 8:**
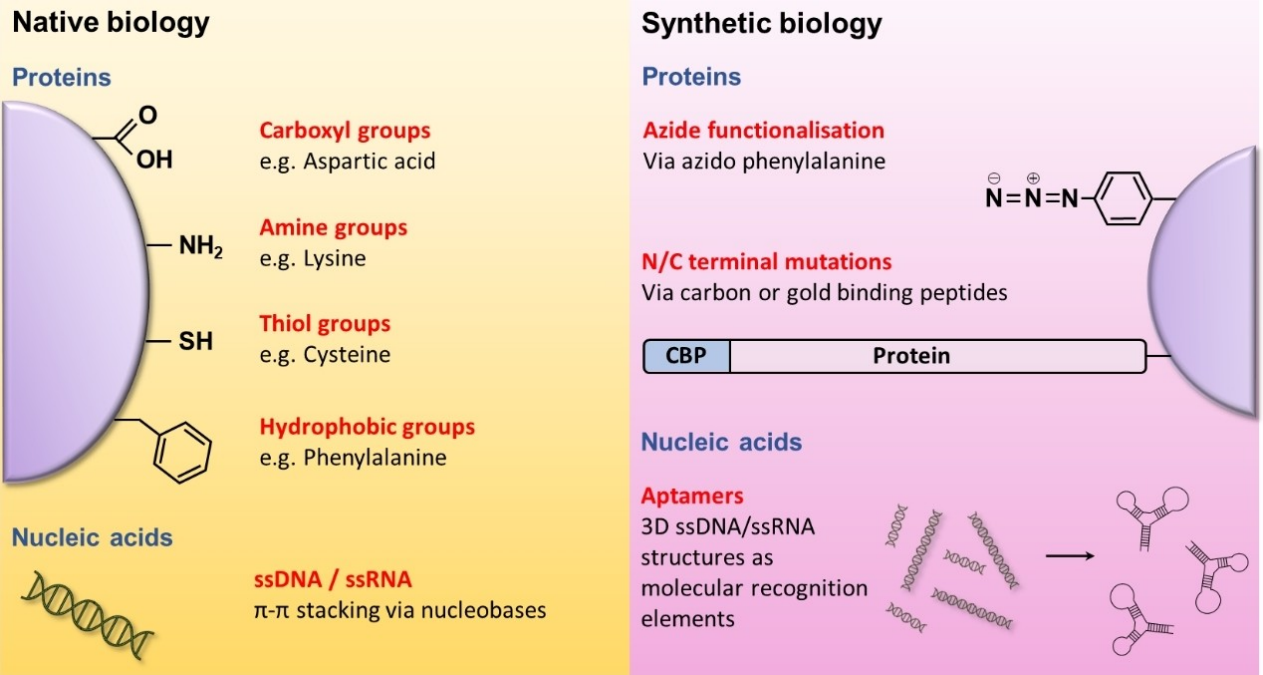
Biomolecule functionalisation strategies. Native biomolecules have a range of functional chemistries at their disposal. Proteins can utilise carboxyl, amine, thiol, and hydrophobic groups as prospective nanocarbon interfacing sites, whilst ssDNA and ssRNA can stack via π–π interactions. Tweaking of biomolecules via synthetic biology introduces new capabilities for proteins, such as azide functionalisation sites and novel N/C terminal mutations to incorporate carbon or gold binding peptides. Aptamers constructed from ssDNA and ssRNA represent its own field of biochemistry but offers huge potential in NC‐FET design.

To consider the conjugation of biomolecules to nanocarbon from a different perspective, one can explore the functional chemistries that biomolecules themselves have to offer. The examples in section 4 predominantly exploit the native functional groups present in protein and nucleic acids, so these will be summarised briefly below. The newest trend, and most likely future direction, that biology is heading in is the use of synthetic biology. Combining in silico modelling with the ever‐evolving knowledge of nanocarbon functionalisation, new chemistries and new blueprints for biomolecule design can be incorporated at the genetic level to tackle some of the problems faced by NC‐FET (Figure 8).

### 5.1 Native biology

When looking to attach biomolecules to nanocarbons, a plethora of functional groups already exist to mediate the conjugation. Proteins, if using the standard genetic code, can have up to twenty different side chains present, supplying a mixture of charged, polar, neutral, and hydrophobic properties. Carboxylic acids can be found on the side chains of aspartic acid and glutamic acid, and can be targeted for carbodiimide activation to covalently crosslink a protein with available amine groups on nanocarbon.[^162^, ^189^] Subsequently in the reverse strategy, amine groups found on the side chains of lysine can be crosslinked to carboxylic acid groups present on nanocarbon via an EDC/NHS coupling reaction.[^46^, ^154^, ^155^] Cysteines offer an alternative conjugation site to gold (electrode and NPs) or via maleimide containing linkers via their thiol group; assuming these are accessible and are not pre‐disposed in structural disulphide bonds or form part of functional centres. Finally, hydrophobic amino acids can mediate protein adsorption through the aromatic side chains of phenylalanine and tryptophan, and aliphatic side chains of isoleucine, leucine, and valine,^[190]^ although as mentioned above, this is generally a weak interaction.

Although native proteins are easier to generate and use, this does come with a lack of control over the conjugation orientation. For example, glutamate and lysine are commonly found on the surface of proteins so you cannot choose to target the side chain of one specific amino acid. Essentially, all accessible side chains can potentially be available for conjugation to the nanocarbon, producing a variety of bound protein orientations. In some cases, this will render the protein useless to the NC‐FET as the analyte binding region may become inaccessible. Cysteine does have an advantage here, as it is the only amino acid offering a free thiol group and is present in proteins at a much lower frequency.^[191]^ Nevertheless, there is still the risk of multiple cysteines in one protein, and thus multiple attachment positions.

Nucleic acids are arguably better suited in their native form for nanocarbon attachment than proteins. Single‐stranded DNA or RNA can naturally wrap SWCNTs or adsorb linearly to graphene through the π–π interactions of the nucleobases,^[169]^ providing an immediate template for hybridisation‐based NC‐FETs.^[138]^ Meanwhile, many of the biofunctionalised NC‐FETs in the literature exploit ssDNA to aid SWCNT dispersion, control protein conjugation sites and mediate the non‐covalent attachment of other biomolecules to the nanocarbon.[^113^, ^170^, ^171^, ^186^] These properties are thus highly desirable and will be used in NC‐FETs for generations to come.

### 5.2 Synthetic biology

Synthetic biology holds considerable promise to further enhance and improve NC‐FET fabrication and operation through the systematic improvement in defined and consistent nanocarbon‐biomolecule interfacing and thus device performance. The examples discussed below offer new perspectives on the biofunctionalisation challenge of nanocarbon.

The first strategy uses azide functionalisation as a novel example of proteins instigating the biochemical link to nanocarbon or a linker. Here, the non‐canonical amino acid p‐azido‐L‐phenylalanine (azF) is incorporated into a recombinant protein at a defined residue through an expanded genetic code.[^192^, ^193^] Phenyl azide chemistry opens up two new routes to nanocarbon functionalisation. The first, mentioned above, uses bioorthogonal click chemistry to attach a receptor protein to linkers (see section 4.3). The second is a direct link between biomolecule and nanocarbon via UV irradiation; a nitrene radical is formed that can directly insert within the nanocarbon framework.[^170^, ^171^, ^174^, ^193^, ^194^, ^195^] This method of conjugation supersedes that of using native biology because the protein can be anchored in a specific orientation, ensuring the analyte‐binding site remains accessible. Gwyther *et al*., recently explored this approach by covalently anchoring super folder green fluorescent protein (sfGFP) to a SWCNT‐FET at two different azide positions. The ability of sfGFP to absorb light and supply electrons to the nanotube channel yielded different device characteristics depending on the protein attachment site. One attachment position established optical memory with sfGFP failing to recycle lost electrons, while the other allowed sfGFP to act as a phototransistor, modulating the current in wavelength‐specific manner.^[196]^ This approach therefore holds great promise for embedding proteins into single‐molecule electronic devices.

Genetic fusions to the N‐ and/or C‐terminus of a protein are standard practise in a biochemical lab, but it is the design and incorporation of carbon or gold binding peptides to these termini which is a novel recent development. Protein adsorption to nanocarbon was at the fundamental core of carbon binding peptide (CBP) design, with research groups exploiting phage display technology^[197]^ to selectively identify peptides and motifs with a strong affinity for nanocarbon.[^198^, ^199^, ^200^] Coyle *et al*., worked to refine these peptides, generating CBPs with differing affinities for sp2 and sp3 carbon. Once fused with an sfGFP reporter protein, they were observed to selectively adsorb to SWCNT sidewalls and ends.^[201]^ This research was then further expanded upon to incorporate a gold binding peptide into the middle of sfGFP, in addition to its C‐terminal CBP,^[202]^ creating a dual‐binding material. Pursuing the chemosensing route, Kuang *et al*. utilised the key advantage CBPs have and simply fused the peptide with a fragment of an insect receptor protein to sense the chemical trinitrotoluene, which allowed sensing in both chemical vapour and liquid‐gated configurations.^[203]^

Aptamers, the final synthetic biology branch, are anticipated to revolutionise the design of biomolecules for nanocarbon functionalisation. Single‐stranded oligonucleotides (including DNA and RNA) undergo a systematic evolution process^[204]^ to generate a sequence that is capable of forming 3D structures to bind with high affinity a target analyte. Ruscito and DeRosa offer an in‐depth review into the science behind aptamers,^[205]^ but their significance in NC‐FET design lies within their ability to mimic proteins without being a protein. Antibodies, for example, are perfectly crafted for ligand recognition, but their large size (10–15 nm)[^206^, ^207^] risks any incoming analyte being beyond the Debye length and thus impossible to sense.^[208]^ This, paired with the ethical costs of antibody production from animals,^[205]^ the functionalisation strategy needed for protein conjugation and the risk of permanent denaturation from changing conditions, offers a challenging proposition for their continued use. Aptamers, being much smaller in size (1–2 nm),^[208]^ cheaper to produce, ease of introducing non‐biological chemistry and arguably easier to functionalise, offer the perfect alternative.^[209]^ Considerations into aptamer use, however, must include the presence of nucleases within clinical samples, as these can degrade the aptamers before any sensing can take place.

## 6 Future Perspectives

From the examples discussed, one can appreciate the extent of design that has gone into the interdisciplinary field of NC‐FETs; but as scientists, we strive to better the technology still. One area that will likely be exploited more is the use of molecular dynamics as a predictive tool. Côte *et al*. performed sophisticated modelling of both DNA and lysozyme in complex with SWCNTs to explore the molecular origin of the electrostatic gating effect.^[210]^ This is of great significance because if we can accurately quantify the gating effect of incoming analytes through modelling, this could be exploited as a viable technique to test potential molecular recognition elements with regards to their orientation, size, and affinity.

Meanwhile, attempts to overcome commercial limitations are continually being developed. Methodological changes to improve fabrication scalability have been reported for NC‐FET arrays[^107^, ^211^] and attempts to outperform conventional silicon complementary MOSFETs have been successful, by reducing the channel length to below 10 nm.[^212^, ^213^] Device uniformity has been targeted through semiconducting CNT dispersion protocols (reaching 99.9 % purity),[^213^, ^214^, ^215^] and functionalisation uniformity is now being addressed to control the number of biomolecules which can interface with an NC‐FET device. Lin *et al*., used a novel DNA‐guiding approach to control the order and extent of covalent functionalisation of CNTs,^[216]^ and one could see this being adapted for biofunctionalisation strategies. Finally, non‐specific analyte binding is being addressed through the straightforward development of high affinity receptors, e. g. aptamers and nanobodies[^204^, ^217^] simplifying protocols by reducing the need for passivation.

After the optimal NC‐FET setups have been established, the next challenge will be to integrate the transistors into a multiplexed array for multianalyte sensing.^[218]^ With input from engineers, logic circuits could be built with integrated decision making, to create small, portable diagnostic devices which provide real‐time monitoring of complex samples. Many applications for multiplexed sensing can be envisaged, for example the time‐sensitive diagnosis of microbial populations in clinical or environmental samples. Traditional techniques, such as microbial culturing, can take days to yield a result,^[219]^ while a point‐of‐care diagnostic test would provide answers within minutes. The usefulness of this, and adaptability of NC‐FETs to target new analytes, will ultimately pave the way for a new generation of bio‐transistors with supreme sensitivity for analyte detection.

## Conflict of interest

The authors declare no conflict of interest.

7

## Biographical Information

*Chang‐Seuk Lee obtained his MSc and PhD in electroanalytical chemistry at the Soonchunhyang University. He is a postdoctoral researcher in the Palma group at Queen Mary University of London (School of Physical and Chemical Sciences, 2021‐Present). His research interests focus on the study of electrical and nanomaterials‐based electrochemical biosensors, from fundamentals to biomedical applications*.

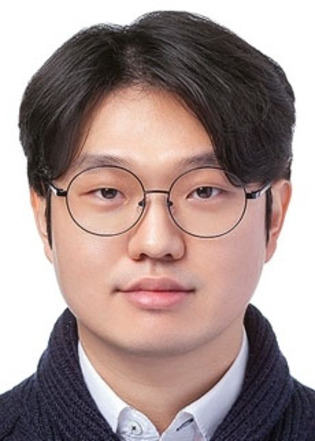

## Biographical Information

*Matteo Palma is Professor of Physical Chemistry and Nanomaterials in the Department of Chemistry, at Queen Mary University of London (UK). He carried out his doctoral studies at the University Louis Pasteur in Strasbourg (France). He then worked as a postdoctoral scientist at Columbia University (New York, USA). Since 2013 he has been a Principal Investigator at Queen Mary University where he leads a research group focusing on the controlled assembly of functional nanostructures. Applications range from studies in the field of (supra)molecular optoelectronics, to biosensing and single‐molecule biological investigations*.

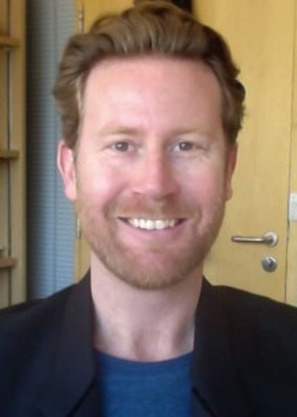

## Biographical Information

*Rebecca Gwyther obtained her MBiol in Biosciences from Durham University in 2018, specialising in molecular biology and biochemistry. She is currently a PhD candidate at Cardiff University, under the guidance of Prof. Dafydd Jones. Her research combines synthetic biology with nanotechnology, to introduce new functional chemistries into proteins and allow them to bind to pristine nanocarbon. The integration of proteins into electrically conducting carbon‐based platforms has allowed her to design field‐effect transistor biosensors for antimicrobial resistance and bio‐optoelectronic transistors*.

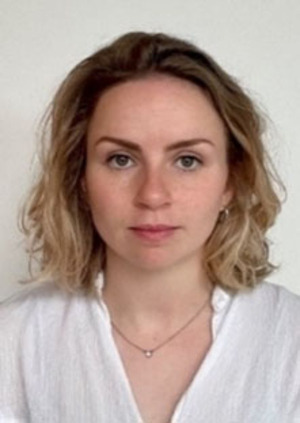

## Biographical Information

*Dafydd Jones obtained his PhD in protein structure and engineering at the University of Cambridge in 1999. He was a postdoctoral researcher at Cambridge before moving to Denmark as a Marie Curie Industrial fellow at Novozymes A/S. He then moved to Cardiff University in 2003 to start his research group, focusing on understanding and applying protein structural and functional plasticity. His group's research is directed at the construction of new protein components and scaffolds, including bionanohybrid systems. His group has developed approaches to engineer proteins to contain new chemistry that allows their precise interfacing with nanomaterials such as nanocarbon*.

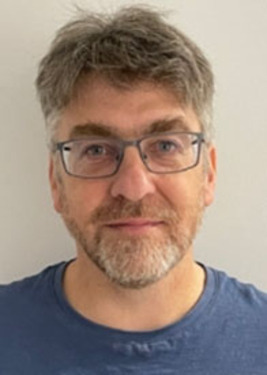

## Biographical Information

*Mark Freeley completed his BA (Moderatorship) in Chemistry in 2012 at Trinity College Dublin. In 2017, he obtained a PhD in chemistry with the Palma group at Queen Mary University of London focusing on self‐assembly and bioconjugation of nanomaterials. He continued as a postdoctoral research assistant in the Palma group with a focus on nanoscale biosensor development*.

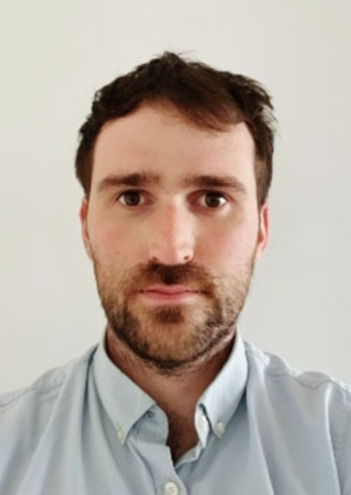

## Data Availability

Data sharing is not applicable to this article as no new data were created or analyzed in this study.
